# Perineal ectopic testis in adult: Experience from tertiary hospital, northern Tanzania

**DOI:** 10.1016/j.ijscr.2022.106817

**Published:** 2022-02-07

**Authors:** Bartholomeo Nicholaus Ngowi, Frank Bright, Jasper Said Mbwambo, Blandina Theophil Mmbaga, Esther Lekei, Mathias Kimolo, Kien Alfred Mteta, Orgeness Jasper Mbwambo

**Affiliations:** a Urology Department, Kilimanjaro Christian Medical Centre, P.O. Box 3010, Moshi, Tanzania; b Urology department, Kilimanjaro Christian Medical University College, P.O. Box 2240, Moshi, Tanzania; c Kilimanjaro Clinical Research Institute, P.O. Box 3010, Moshi, Tanzania

**Keywords:** Case report, Orchidopexy, Perineal testis, Tanzania

## Abstract

**Introduction and importance:**

Perineal ectopic testis is a rare congenital abnormality mostly diagnosed during childhood period. The diagnosis can be easily reached by physical examination. The treatment of choice is either orchidopexy or orchiectomy through scrotal or inguinal approach.

**Case presentation:**

We report a case of 30 year old male with painful perineal mass and empty right hemiscrotum. Ultrasound of the mass was done prior to operative procedure and the ectopic testis was the working diagnosis ever since. At exploration there was no abnormality found on the testis; thus, orchidopexy was done with uneventful postoperative period.

**Clinical discussion:**

Ectopic testis is rare congenital anomaly with perineal ectopic testis accounting for only 1% of all cases. Most cases of ectopic testis are diagnosed during childhood period. Our case presented at the age of 30 years, the reason could be either late diagnosis by physician or lack of insight of parents/care takers or both. The late presentation might pose a diagnostic challenge but also the testis may be atrophic or undergo malignant changes. In this case the testis was normal and therefore it was successfully relocated surgically through trans-inguinal approach.

**Conclusion:**

It is suggested to make perineal ectopic testis one of the differential diagnoses on painful perineal swelling without ipsilateral testis on any man.

## Highlights

• Perineal ectopic testis is a very rare anomaly that represents a true ectopic location of the tests.
• It is more rare in adults and young boys.
• Diagnosis depends on level of suspicious index as it is mostly clinical.
• Any man presenting with painful perineal swelling in the setting of absent testis should be regarded as having perineal ectopic testis until proved other wise.
• Trans-inguinal orchidopex offers a safe way of managing perineal ectopic testis as the testis is usually of normal size and the spermatic cord is of adequate length even in adults.

## 1 Background

Testicular maldescent is a common congenital genital abnormality in boys [1], [2]. Testicular maldescent may be undescended with the testis being found anywhere along its normal path of descent or it may land in an ectopic sites outside the normal path of descent and in this case referred to as ectopic testis [1], [3], [4]. Undescended testis is the commonest type of testicular maldescent while ectopically located testis being rare [1], [4], [5] The aetiology of ectopic testis is not very clear. Some postulated that ectopic testis is the result of error in the gubernacular as a guide of the testicular descent [2], [3], [5], [6], [7]. Hereditary factors have also been implicated as a risk for ectopic testis (2). The common location of ectopic testis in descending order is superficial inguinal pouch, perineum, femoral canal, contralateral hemiscrotum and prepenile area (2). Perineal ectopic testis is a rare congenital anomaly that represents a true form of ectopic testis with the prevalence of only 0.2% of all undescended testis [6], [8]. The diagnosis is clinical and the typical finding include and empty scrotum and palpable testes in the ectopic sites [2], [4], [5], [6] Moreover, ultrasonography and tomography are rarely required (2).

We present a case of 30-year-old male who presented to Kilimanjaro Christian Medical Centre (KCMC) hospital with right-sided perineal ectopic testis that was managed with orchidopexy successfully.

This work has been reported in line with the SCARE 2020 criteria (9).

## 2 Case presentation

**Fig. 1:**
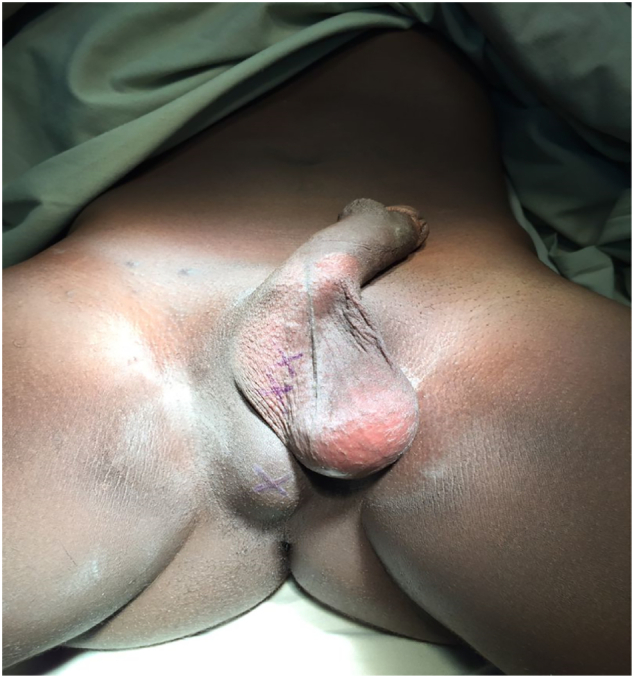
Area marked xx shows an underdeveloped empty left hemiscrotum, x is the ovoid perineal swelling that was finally found to be the left testis.

**Fig. 2:**
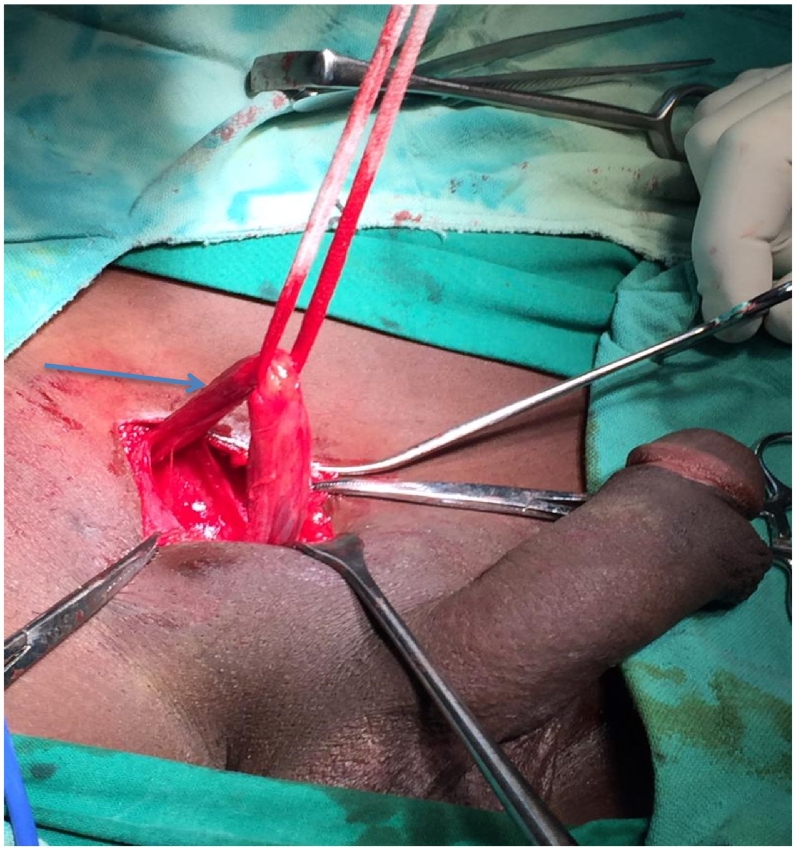
showing left inguinal incision with the identification of the left spermatic cord (arrow).

**Fig. 3:**
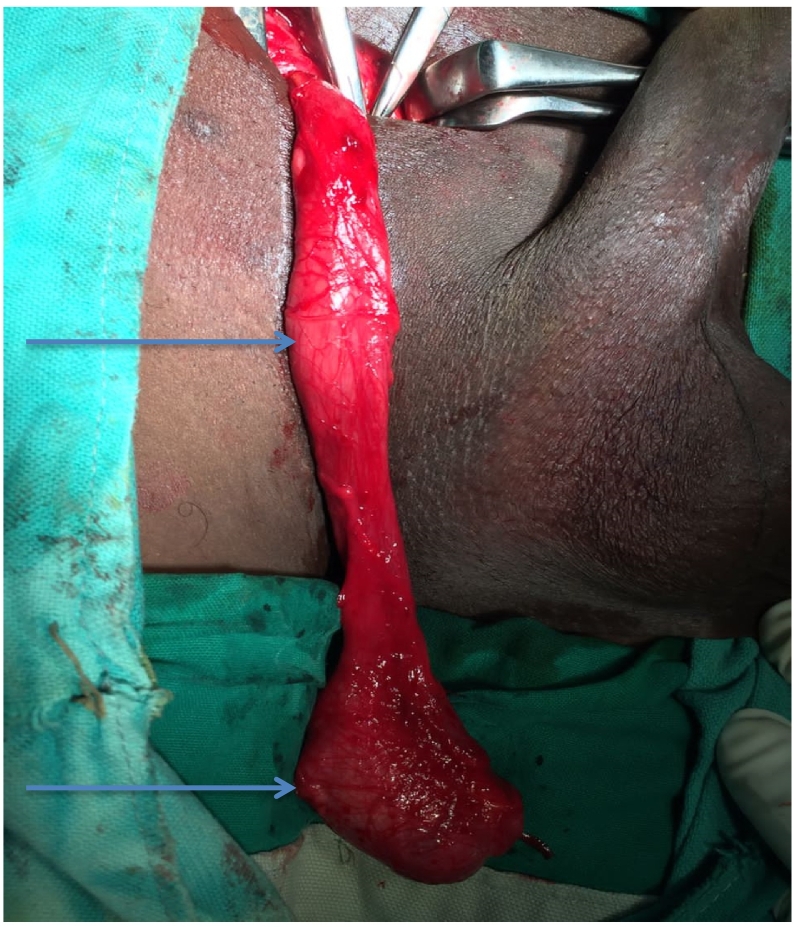
shows an extra long left spermatic cord (top arrow) and normal sized left testis (bottom arrow).

**Fig. 4:**
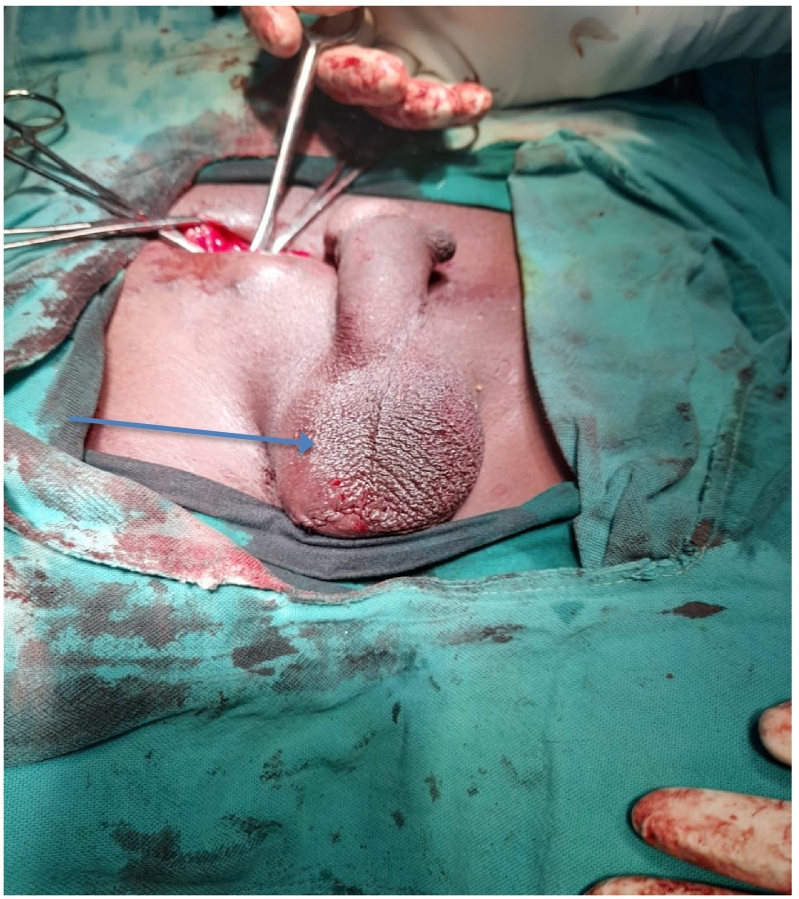
showing the left hemiscrotum containing the left testis following orchidopexy (arrow).

A 30-year-old male, presented at KCMC hospital with history of absence of the left testis in the scrotum since birth. Later on when he was 15 years, he noticed a perineal swelling that was progressively increase in size. The swelling was initially painless but started to be painful when sitting and it was progressively increasing in severity with time for about one year. However, there was no history of increase in size or pain on standing position, fevers or nausea/vomiting. The patient reported to have no voiding or sexual function problems. He further denied history of any surgery or being on any medication regularly as well as history of similar problem in the family. Upon examination he was noted to have hypoplastic right hemiscrotum (Fig. 1) while the left testis was normal in size, shape and texture. There was well-circumscribed oval shaped mass on the right side of the perineum measuring about 1 × 1 centimetre. The mass was non-tender, freely mobile, firm in consistence and overlying skin was normal in colour and pinchable (Fig. 1). We had a provisional diagnosis of perineal ectopic testis with differential diagnosis of lipoma, sebaceous cyst, dermoid cyst and abscess. Ultrasound of the perineal mass revealed a homogenous, well vascularised testicular like structure with a volume of 6.5 cc, the left testis had normal echo texture measuring 7.6 cc. The patient was planned for open orchidopexy through inguinal approach (Fig. 2). Intra operatively the spermatic cord was identified on the inguinal area (Fig. 2) and followed downward where it was found to be attached to the perineal structure. The cord and perineal testis were dissected off and delivered out. The spermatic cord was noted to be longer than normal, however the testis and epididymis were normal with no associated hernia (Fig. 3). That testis was re-positioned in the sub-dartos pouch on the right hemiscrotum (Fig. 4). Post operatively he faired well and at two month post operatively was found to have no problem and the wound had healed well.

## 3 Discussion

Testicular descent is a complex process that is influenced by several factors including hormonal and mechanical factors (2). The most important mechanical factor, gubernaculum begins as a mesenchyme band that originates on the lower pole of the testis and inserts in the scrotum. It is though that gubernaculum guides the testis into the scrotum through various mechanisms that include, traction, muscular contraction and differential growth around the fixed point.

Abnormality in the process of decent can result into abnormally located testis along the normal part of descent referring to as undescended testis or location of the testis outside its normal path of descent which is called ectopic testis (2). Ectopic testis is a rare congenital anomaly, with the tests being located in various ectopic places including superficial inguinal pouch in 75% and perineal location in 1% of all cases. John Hunter first described Perineal ectopic testis back in year 1786 [2], [6], [10] Diagnosis is based on history and physical examination (2) as in our case.

In children, perineal ectopic testis may be found on the lower part of the scrotum (2). Our case was a young adult with the testis fully descended to the perineal ectopic site that is very far from the scrotum. Most cases of ectopic testis present early in childhood [2], [3], [4], [10] while adult presentation is rare (1). Although we correctly diagnosed this case, we experienced a big diagnostic challenge due to the late presentation. Factors such as lack of new born examination by clinicians, inadequate parental education and neglected or delayed referral have been found to be the main reasons for late presentation of undescended testis [11], [12]. Since ectopic testis and undescended testis are all testicular maldescent, similar factors might have contributed to the late presentation of the index case.

Delay in diagnosis and treatment may lead into testicular atrophy, trauma, cancer, torsion and infertility in cases of bilateral ectopia [1], [2], [7], [10]. Our patient was experiencing pain probably due to trauma as a result of compression especially on siting position. The recommended treatment options are surgical relocation of the testis into the scrotum for normal sized testis and orchiectomy with fixation of the contralateral testis if the testis is severely atrophied [1], [2], [6], [10]. The undescended testis is not operated before the age of 6 months, however for ectopic testis it should be operated before that age (5). In our case there was a delay as the case was diagnosed at the age of 30 years. However, the testis was normal in size, shape and consistence to gather with echo-texture and therefore orchidopexy was performed. Surgical approaches are either inguinal or scrotal approach. The former being suitable as there may be associated hernia [2], [5], [7]. In this case there was no evidence of hernia or persistence of processus vaginalis. Like our case, the testis can be easily positioned into the scrotum as a result of adequate spermatic cord length and its associated vessels [2], [7]. The index patient had extremely long spermatic cord and the associated structures. Long-term follow up is recommended as the outcome is very similar to other forms of testicular maldescent [5], [7]. In this case, ultrasound pictures of the perineal ectopic testis were not available at the time of writing this case, this could have supported our case description. Moreover, we could not establish the exactly reason for the late presentation of the index case which could have helped in the prevention.

## 4 Conclusions

Perineal testis is very rare congenital anomaly of boys. The diagnosis is clinical based on the absence of testis in the scrotum and perineal location of oval shaped structure. Orchidopexy is the treatment of choice in cases of normal testicular size and consistence that gives good immediate postoperative outcome. Any man presenting with painful perineal swelling in the absence of testis in the scrotum should be examined thoroughly including the perineum to exclude possibility of perineal ectopic testis.

## Funding

There was no any financial support received from individual or company.

## Ethical approval

Ethical standards were reviewed and approved by urology team.

## Consent

Written informed consent was obtained from the patient for publication of this case report and accompanying images. A copy of the written consent is available for review by the Editor-in-Chief of this journal on request.

## Research registration

N/A

## Guarantor

Bartholomeo Nicholaus.

## CRediT authorship contribution statement

BNN, JSM, FB, BTM and KAM reviewed the patient and planned the treatment. BNN, MM and EL operated the patient and followed him post operatively. BNN and OJM conceptualized and prepared the first manuscript. All authors read and approved the final manuscript.

## Declaration of competing interest

All authors declare that they have no conflict of interest regarding the information presented in this case report.
